# TDAE Strategy in the Benzoxazolone Series: Synthesis and Reactivity of a New Benzoxazolinonic Anion

**DOI:** 10.3390/molecules20011262

**Published:** 2015-01-14

**Authors:** Aïda R. Nadji-Boukrouche, Omar Khoumeri, Thierry Terme, Messaoud Liacha, Patrice Vanelle

**Affiliations:** 1 Département de Génie des Procédés, Université 8 mai 1945 Guelma, BP 401, Guelma 24000, Algeria; E-Mail: nadji_aida@yahoo.fr; 2 Laboratoire de Synthèse et de Biocatalyse Organique (LSBO), Faculté des Sciences, Université Badji Mokhtar-Annaba, BP 12 El-Hadjar, Annaba 23000, Algeria; E-Mail: m_liacha@yahoo.fr; 3 Aix-Marseille Université, CNRS, Institut de Chimie Radicalaire ICR, UMR 7273, Laboratoire de Pharmaco-Chimie Radicalaire, Marseille 13385, France; E-Mails: omar.khoumeri@univ-amu.fr (O.K.); thierry.terme@univ-amu.fr (T.T.)

**Keywords:** TDAE, benzoxazolone, benzoxazolinonic anion, benzylic alcohols, oxiranes

## Abstract

We describe an original pathway to produce new 5-substituted 3-methyl-6-nitro-benzoxazolones by the reaction of aromatic carbonyl and α-carbonyl ester derivatives with a benzoxazolinonic anion formed exclusively via the TDAE strategy.

## 1. Introduction

Many benzoxazolinone derivatives have been described in therapeutics as possessing a wide variety of pharmacological activities [1,2,3,4,5,6,7,8,9,10]. Indeed, the clinical applications of this template are very broad, and range from analgesic anti-inflammatory compounds to antipsychotic and neuroprotective anticonvulsant compounds [11]. Several potentially useful drugs and pharmacological tools based on these pharmacophores have been developed in recent years [12,13,14,15,16].

Tetrakis(dimethylamino)ethylene (TDAE) is a reducing agent which reacts with halogenated derivatives to generate an anion under mild conditions via two sequential transfers of one electron [17,18,19]. Through this strategy, we have developed many reactions between nitrobenzylic substrates and a series of electrophiles such as aldehydes, ketones, α-ketoesters, α-ketolactams and ketomalonates leading to corresponding alcohol adducts [20,21,22,23]. This reactivity was recently extended using original heterocyclic carbaldehydes as electrophiles. The reactions led to the expected products, while at the same time bringing to light a new and original reactivity and enabling us to define some limitations of this strategy [24]. Moreover, we reported the reactions of dihalo- and trihalomethyl heterocyclic derivatives with aromatic aldehydes in the presence of TDAE, providing a mixture of *cis*/*trans* isomers of oxiranes and α-haloketone derivatives, respectively [25,26]. In the same context, the expected alcohols and oxiranes were obtained in good yields in the quinonic series [27].

In continuation of our research program centered on the design and synthesis of novel bioactive molecules [28,29,30,31,32], we report herein the preparation of 5-(bromomethyl)-3-methyl-6-nitrobenzoxazolone (**2**) and 5-(dibromomethyl)-3-methyl-6-nitrobenzoxazolone (**3**) and the study of their reactivity with various aromatic carbonyl and α-carbonyl ester derivatives using the TDAE methodology.

## 2. Results and Discussion

### 2.1. Synthesis of Mono and Dibromide Substrates

We prepared 5-(bromomethyl)-3-methyl-6-nitrobenzoxazolone (**2**) and 5-(dibromomethyl)-3-methyl-6-nitrobenzoxazolone (**3**) [33] in four and five steps, respectively. The condensation of 2-amino-4-methylphenol with urea was inspired by a previously described method [34,35]. After methylation using dimethyl sulfate, the nitration of the obtained 3,5-dimethylbenzoxazolone by action of a mixture of nitric and sulfuric acids afforded 3,5-dimethyl-6-nitrobenzoxazolone (**1**) in 88% yield.

**Scheme 1:**
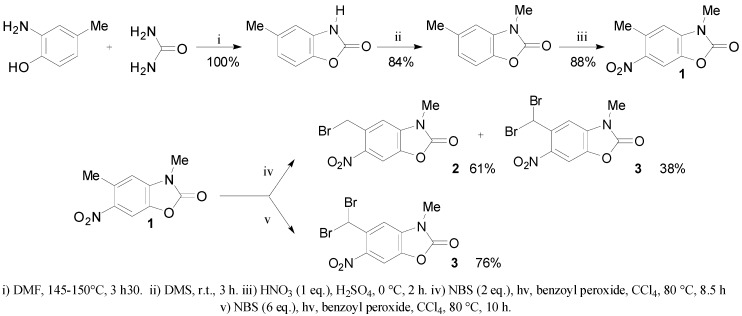
Synthesis of 5-(bromomethyl)-3-methyl-6-nitrobenzoxazolone (**2**) and 5-(dibromomethyl)-3-methyl-6-nitrobenzoxazolone (**3**).

The bromination of **1** with 2 equivalents of *N*-bromosuccinimide in refluxing CCl_4_ for 8.5 h gave 5-(bromomethyl)-3-methyl-6-nitrobenzoxazolone (**2**) in 61% yield, accompanied by 5-(dibromomethyl)-3-methyl-6-nitrobenzoxazolone (**3**) in 38% yield. However, the preparation of this latter compound was optimized (76%) using 6 equivalents of *N*-bromosuccinimide in refluxing CCl_4_ for 10 h (Scheme 1).

### 2.2. TDAE Reactivity of 5-(Bromomethyl)-3-methyl-6-nitrobenzoxazolone (**2**)

**Scheme 2:**
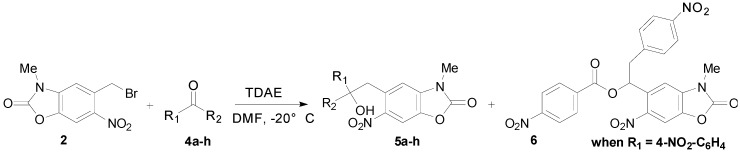
TDAE reactivity of 5-(bromomethyl)-3-methyl-6-nitrobenzoxazolone (**2**) with aromatic carbonyl and some α-keto-ester derivatives **4a**–**h**.

The reaction of 5-(bromomethyl)-3-methyl-6-nitrobenzoxazolone (**2**) with 3 equivalents of various aromatic carbonyl and α-carbonyl ester derivatives **4a**–**j** in the presence of TDAE at −20 °C for 1 h, followed by 2 h at room temperature (r.t.) led to the corresponding alcohol derivatives **5a**–**j** in moderate to good yields (31%–72%) as shown in Table 1 and Scheme 2.

**Table 1:** Reaction of bromide **2** with aromatic carbonyl and α-carbonyl ester derivatives using TDAE ^a^.

Entry ^a^	Aromatic Carbonyl	*R*_1_	*R*_2_	Product Number	Yield (%) ^b^
**1**	4-Nitrobenzaldehyde	4-NO_2_-C_6_H_4_	H	**5a**	52
**2**	4-Bromobenzaldehyde	4-Br-C_6_H_4_	H	**5b**	49
**3**	4-Cyanobenzaldehyde	4-CN-C_6_H_4_	H	**5c**	31
**4**	2-Nitrobenzaldehyde	2-NO_2_-C_6_H_4_	H	**5d**	44
**5**	2-Bromobenzaldehyde	2-Br-C_6_H_4_	H	**5e**	49
**6**	3-Bromobenzaldehyde	3-Br-C_6_H_4_	H	**5f**	43
**7**	Ethyl glyoxylate	CO_2_C_2_H_5_	H	**5g**	72
**8**	Diethyl ketomalonate	CO_2_C_2_H_5_	CO_2_C_2_H_5_	**5h**	62

Notes: ^a^ All the reactions were performed using 3 equivalents of aromatic carbonyl **4a**–**h**, 1 equivalent of bromide **2** and 1 equivalent of TDAE in anhydrous DMF stirred at −20 °C for 1 h and then warmed to rt for 2 h; ^b^ % Yield relative to bromide **2**.

The reaction of substrate **2** with the aromatic aldehydes **4a**–**f** under TDAE-initiated conditions furnished the expected alcohols **5a**–**f** in moderate to good yields. The best yield (52%) was obtained with *p*-nitrobenzaldehyde (**4a**). Unexpectedly, *o,p*-bromobenzaldehyde (**4e**,**4b**) gave the same yield (49%), while *o*-nitrobenzaldehyde (**4d**) and *m*-bromobenzaldehyde (**4f**) gave approximately the same yield (44% and 43%, respectively). Notably, with *p*-nitrobenzaldehyde (**4a**) we observed 23% of the ester **6**. According to a recent mechanistic study [36], the formation of the unexpected ester derivative **6** may be explained by an electron transfer in a primary step between 4-nitrobenzaldehyde (**4a**) as acceptor and TDAE as donor.

*p*-Cyanobenzaldehyde (**4c**) produced a moderate yield (31%). The formation of these alcohol derivatives may be explained by nucleophilic addition of benzazolinonic carbanions formed by the action of TDAE with 5-(bromomethyl)-3-methyl-6-nitrobenzoxazolone (**2**) on the carbonyl group of the corresponding aldehyde. In summary, the difference in yields does not appear to be totally explained by electronic effects: the halogen groups furnished approximately the same yields in either position. With nitrobenzaldehydes, steric hindrance could explain the difference between *o*- and *p*-nitrobenzaldehyde yields (44% *versus* 52%).

It is important to note that in the reactions of substrate **2** with the electrophiles **4b**–**f**, we observed the unavoidable formation of the reduction product **1** [37]. Extending the reaction times to 8 h at ambient temperature increases its percentage, but decreases the yield of alcohol. On the other hand, after 4 h of reaction, the percentage of reduction product decreases at the same time as that of the alcohol: in this case we also observed traces of the dimerization of substrate **2**.

**Scheme 3:**
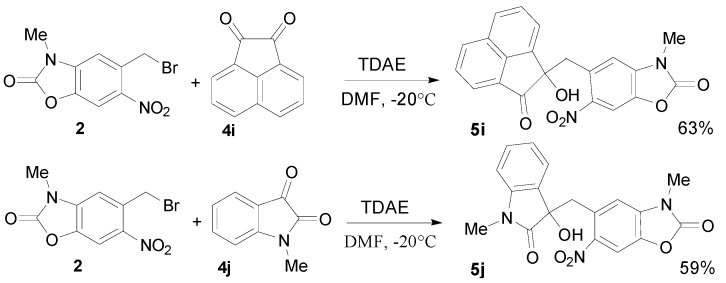
TDAE reactivity of the 5-(bromomethyl)-3-methyl-6-nitrobenzoxazolone (**2**) and α-diketone and α-ketolactam derivatives **4i**–**j**.

Moreover, after the reaction with aromatic aldehydes, we investigated the reaction of **2** with α-keto-ester derivatives such as ethyl glyoxylate (**4g**), diethyl ketomalonate (**4h**), acenaphtenedione (**4i**) and 1-methylisatin (**4j**) in the presence of TDAE. The reactions with these electrophiles furnished the corresponding hydroxyl derivatives **5i**–**j** in good yields (59%–63%), as shown in Table 1 and Scheme 3.

### 2.3. TDAE Reactivity of 5-(Dibromomethyl)-3-methyl-6-nitrobenzoxazolone (**3**)

**Scheme 4:**
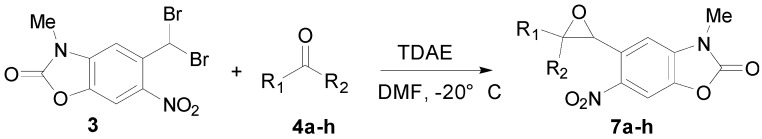
TDAE reactivity of 5-(dibromomethyl)-3-methyl-6-nitrobenzoxazolone (**3**) with aromatic carbonyl and some α-keto-ester derivatives **4a**–**h**.

The optimized protocol of the dibromomethyl derivative **3**, was defined with 3 equivalents of aromatic carbonyls **4a**–**h**, 1 equivalent of 5-(dibromomethyl)-3-methyl-6-nitrobenzoxazolone (**3**) and 1.5 equivalents of TDAE in anhydrous DMF, for 1 h at −20 °C followed by 2 h at r.t. The reactions led to a mixture of *cis*/*trans* isomers of the corresponding oxiranes **7a**–**h** in moderate to good yields as reported in Table 2 (Scheme 4). The formation of these oxiranes may be explained by nucleophilic addition of a α-bromocarbanion, formed by the action of TDAE with 5-(dibromomethyl)-3-methyl-6-nitrobenzoxazolone (**3**), on the carbonyl group of aldehydes **4a**–**h** followed by an intramolecular nucleophilic substitution [26].

In the case of the nitroaromatic aldehydes, steric hindrance could explain the yield difference between *o*- and *p*-nitrobenzaldehyde (46% and 63%). However, this effect disappears in the *o*-bromo-substituted aldehyde which gave 64% of the corresponding oxirane, the *p*- and *m*- substituted aldehydes with 55 and 48% yields respectively. *p*-Cyanobenzaldehyde gave the expected oxirane in good yield (72%).

**Scheme 5:**
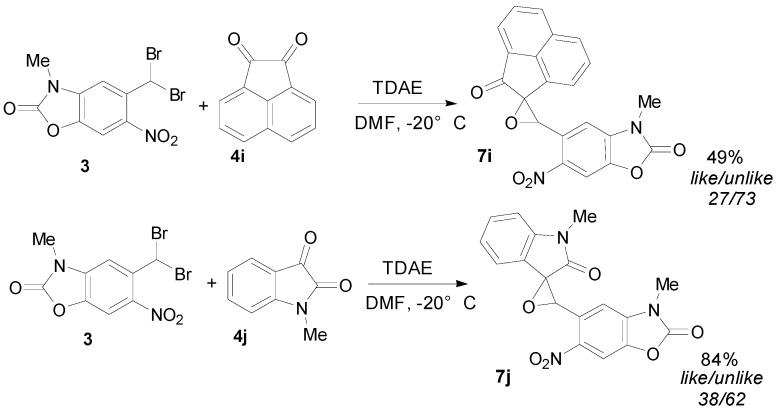
TDAE reactivity of 5-(dibromomethyl)-3-methyl-6-nitrobenzoxazolone (**3**) and α-keto-ester derivatives **4i**–**j**.

Under the same experimental conditions, we studied the reaction of derivative **3** with α-keto-ester derivatives **4g**–**h** as reported in Table 2 (Scheme 4). Only the *trans* isomers of the oxiranes **7g** and **7h** were obtained in 26% and 37% yields, respectively, with ethyl glyoxylate (**4g**) and diethyl ketomalonate (**4h**). Otherwise, acenaphtenedione (**4i**) and methyl isatin (**4j**) furnished mixtures of *like*/*unlike* original stereoisomers **7i** and **7j**, respectively, in good yields (Scheme 5). The diastereoisomers were separable, and their configuration was identified by NMR-analysis from the γ-left effect, as previously described [26,38].

**Table 2:** Reaction of dibromide **3** with aromatic carbonyl and α-carbonyl ester derivatives using TDAE ^a^.

Entry ^a^	Aromatic Carbonyl	*R*_1_	*R*_2_	Oxirane	Cis/Trans Isomers % ^b^	Yield (%) ^c^
**1**	4-Nitrobenzaldehyde	4-NO_2_-C_6_H_4_	H	**7a**	15/85	63
**2**	4-Bromobenzaldehyde	4-Br-C_6_H_4_	H	**7b**	7/93	55
**3**	4-Cyanobenzaldehyde	4-CN-C_6_H_4_	H	**7c**	15/85	72
**4**	2-Nitrobenzaldehyde	2-NO_2_-C_6_H_4_	H	**7d**	32/68	46
**5**	2-Bromobenzaldehyde	2-Br-C_6_H_4_	H	**7e**	19/81	64
**6**	3-Bromobenzaldehyde	3-Br-C_6_H_4_	H	**7f**	7/93	48
**7**	Ethyl glyoxylate	CO_2_C_2_H_5_	H	**7g**	0/100	26
**8**	Diethyl ketomalonate	CO_2_C_2_H_5_	CO_2_C_2_H_5_	**7h**	0/100	37

Notes: ^a^ All the reactions were performed using 3 equivalents of aromatic carbonyl **4a**–**h**, 1 equivalent of dibromide **3** and 1.5 equivalent of TDAE in anhydrous DMF stirred at −20 °C for 1 h and then warmed to r.t for 2 h; ^b^ % isomers determined on ^1^H-NMR measurements from the crude product; ^c^ % yield relative to dibromide **3**.

The relative *cis*/*trans* percentages of oxirane isomers reported in Table 2 showed that the stereoselectivity of these reactions is not only sensitive to steric hindrance, but it also depends on the nature of the electrophile substituents. The reactions with bromo-substituted aldehydes in either position were more selective than with nitro-substituted aldehydes. The same percentages of *cis*/*trans* isomers were previously reported with *p*-nitro- and cyanobenzaldehyde. However, the reactions with ethyl glyoxylate and diethyl ketomalonate were the most selective. Moreover, stereoselectivity was recorded in the mixtures of *like*/*unlike* original stereoisomers with methyl isatin and acenaphtenedione.

## 3. Experimental Section

### 3.1. General Information

Melting points were determined on a Buchi capillary melting point apparatus and are uncorrected. Elemental analyses were performed by the Centre de Microanalyses of the University of Aix-Marseille. Both ^1^H- (200 MHz) and ^13^C-NMR (50 MHz) spectra were determined on a Bruker AC 200 spectrometer. The ^1^H chemical shifts are reported as parts per million downfield from tetramethylsilane (Me_4_Si), and the ^13^C chemical shifts were referenced to the solvent peaks: CDCl_3_ (76.9 ppm) or Me_2_SO-*d*_6_ (39.6 ppm). Absorptions are reported using the following notation: s, singlet; d, doublet; t, triplet; q, quartet; m, a more complex multiplet or overlapping multiplets. The following adsorbents were used for column chromatography: silica gel 60 (Merck, Darmstadt, Germany, particle size 0.063–0.200 mm, 70–230 mesh ASTM). TLC was performed on 5 cm × 10 cm aluminium plates coated with silica gel 60 F-254 (Merck) in an appropriate solvent. 3,5-Dimethyl-6-nitrobenzoxazolone (**1**) was synthesized in three steps: condensation of 2-amino-4-methylphenol with urea [34], methylation using dimethyl sulfate and nitration by action of a mixture of nitric and sulfuric acids.

### 3.2. Synthesis of Substrates **1**–**3**

*3,5-Dimethyl-6-nitrobenzo[d]oxazol-2(3H)-one* (**1**): yellow solid; mp 159 °C (EtOH); ^1^H-NMR (CDCl_3_): δ 2.70 (s, 3H, CH_3_), 3.45 (s, 3H, NCH_3_), 6.87 (s, 1H, CH), 7.95 (s, 1H, CH). ^13^C-NMR (CDCl_3_): δ 21.6 (CH_3_), 28.5 (NCH_3_), 107.3 (CH), 110.7 (CH), 132.2 (C), 135.7 (C), 140.2 (C), 143.5 (CNO_2_), 154.3 (CO). Anal. Calcld. for C_9_H_8_N_2_O_4_ (208.17): C, 51.93; H, 3.87; N; 13.46. Found: C, 52.34; H, 3.95; N, 13.40.

5-(Bromomethyl)-3-methyl-6-nitrobenzo[*d*]oxazol-2(3*H*)-one (**2**) and 5-(dibromomethyl)-3-methyl-6-nitrobenzo[*d*]oxazol-2(3*H*)-one (**3**) were prepared according to a previously described method [27].

*5-(Bromomethyl)-3-methyl-6-nitrobenzo[d]oxazol-2(3H)-one* (**2**): yellow solid (EtOH); mp 120 °C; ^1^H-NMR (CDCl_3_): δ 3.49 (s, 3H, CH_3_), 4.91 (s, 2H, CH_2_Br), 7.14 (s, 1H, CH), 7.99 (s, 1H, CH). ^13^C-NMR (CDCl_3_): δ 28.7 (NCH_3_), 29.3 (CH_2_Br), 108.0 (CH), 110.6 (CH), 131.1 (C), 136.1 (C), 141.7 (C), 142.6 (CNO_2_), 153.9 (CO). Anal. Calcld for C_9_H_7_BrN_2_O_4_ (287.07): C, 37.66; H, 2.46; N; 9.76. Found: C, 38.48; H, 2.58; N, 9.88.

*5-(Dibromomethyl)-3-methyl-6-nitrobenzo[d]oxazol-2(3H)-one* (**3**): yellow solid (EtOH); mp 134 °C; ^1^H-NMR (CDCl_3_): δ 3.55 (s, 3H, NCH_3_), 7.55 (s, 1H, CHBr_2_), 7.75 (s, 1H, CH), 7.78 (s, 1H, CH). ^13^C-NMR (CDCl_3_): δ 29.0 (NCH_3_), 34.4 (CHBr_2_), 106.2 (CH), 110.7 (CH), 134.3 (C), 136.5 (C), 139.0 (C), 142.0 (CNO_2_), 153.6 (CO). Anal. Calcld for C_9_H_7_BrN_2_O_4_ (365.96): C, 29.54; H, 1.65; N; 7.65. Found: C, 29.59; H, 1.67; N, 7.69.

### 3.3. General Procedure for the Reaction of **2** and Aromatic Carbonyl Derivatives **4a**–**f**, α-Carbonyl Ester **4g**, Ketomalonate **4h**, Acenaphtenedione **4i** and Ketolactam **4j** Using TDAE

A solution of **2** (0.5,1.74 mmol) in anhydrous DMF (10 mL) and the corresponding carbonyl derivative **4a**–**j** (5.22 mmol, 3 equivalents) were placed under nitrogen at −20 °C in a two-necked flask equipped with a silica-gel drying tube and a nitrogen inlet. The solution was stirred and maintained at this temperature for 30 min and then the TDAE (0.41 mL, 1.74 mmol, 1 equivalent) was added dropwise via a syringe. A red color immediately developed with the formation of a fine white precipitate. The solution was vigorously stirred at −20 °C for 1 h and then warmed to r.t. for 2 h. After this time TLC analysis (dichloromethane) clearly showed that **2** was totally consumed. The orange-red turbid solution was filtered (to remove the octamethyloxamidinium dibromide) and hydrolyzed with 80 mL of H_2_O. The aqueous solution was extracted with toluene (3 × 40 mL), the combined organic layers washed with H_2_O (3 × 40 mL) and dried over MgSO_4_. Evaporation of the solvent left an orange viscous liquid as crude product. Purification by silica gel chromatography and recrystallization in ethyl alcohol gave the corresponding products.

*5-(2-Hydroxy-2-(4-nitrophenyl)ethyl)-3-methyl-6-nitrobenzo[d]oxazol-2(3H)-one* (**5a**): Brown solid; mp 233 °C; ^1^H-NMR (DMSO-*d*_6_): δ 3.37 (s, 3H, NCH_3_), 3.17–3.33 (m, 2H, 2 × CH), 4.92–5.01 (m, 1H, 1H, CH), 5.67 (bs, 1H, OH), 7.33 (s, 1H, CH), 7.61 (d, *J* = 8,5 Hz, 2H, 2 × CH), 8.00 (s, 1H, CH), 8.21 (d, *J* = 8.5 Hz, 2H, 2 × CH). ^13^C-NMR (DMSO-*d*_6_): δ 28.9 (NCH_3_), 42.2 (CH_2_), 72.1 (CH), 106.7 (CH), 112.5 (CH), 123.6 (2 × CH), 127.0 (2 × CH), 131.2 (C), 135.8 (C), 140.2 (C), 144.1 (C), 146.7 (C), 153.2 (C), 154.3 (CO). HRMS (EI): calcd for C_16_H_13_N_3_O_7_ (M^+^) 337.1092, found 337.1092.

*5-(2-(4-Bromophenyl)-2-hydroxyethyl)-3-methyl-6-nitrobenzo[d]oxazol-2(3H)-one* (**5b**): Brown solid; mp 213 °C; ^1^H-NMR (CDCl_3_): δ 2.13 (d, *J* = 3.2 Hz, 1H, OH), 3.13 (dd, *J* = 13.7 Hz, *J* = 9.1 Hz, 1H, CH), 3.43 (s, 3H, NCH_3_), 3.50 (dd, *J* = 13.7 Hz, *J* = 3.7 Hz, 1H, CH), 5.06 (dd, *J* = 9.1 Hz, *J* = 3.7 Hz, 1H, CH), 6.85 (s, 1H, CH), 7.33 (d, *J* = 8.4 Hz, 2H, 2 × CH), 7.52 (d, *J* = 8.4 Hz, 2H, 2 × CH), 7.94 (s, 1H, CH). ^13^C-NMR (CDCl_3_): δ 28.6 (NCH_3_), 43.7 (CH_2_), 73.5 (CH), 107.5 (CH), 111.9 (CH), 121.7 (C), 127.3 (2 × CH), 131.7 (2 × CH), 135.6 (C), 140.8 (C), 142.7 (CH), 144.0 (C), 154.3 (CO). C-NO_2_ was not observed under these experimental conditions. Anal. Calcld for C_16_H_13_BrN_2_O_5_ (393.19) C, 48.88; H, 3.33; N; 7.12. Found: C, 48.91; H, 3.39; N, 7.19.

*4-(1-Hydroxy-2-(3-methyl-6-nitro-2-oxo-2,3-dihydrobenzo[d]oxazol-5-yl)ethyl)benzonitrile* (**5c**): Yellow solid; mp 213 °C; ^1^H-NMR (CDCl_3_): δ 2.27 (d, *J* = 3.0 Hz, 1H, OH), 3.05 (dd, *J* = 13.5 Hz, *J* = 9.4 Hz, 1H, CH), 3.46 (s, 3H, NCH_3_), 3.57 (dd, *J* = 13.5 Hz, *J* = 2.6 Hz, 1H, CH), 5,15 (dd, *J* = 9.4 Hz, *J* = 2.6 HZ, 1H, CH), 6.93 (s, 1H, CH), 7.61 (d, *J* = 8.3 Hz, 2H, 2 × CH), 7.70 (d, *J* = 8.3 Hz, 2H, 2 × CH); 7.98 (s,1H, CH). ^13^C-NMR (CDCl_3_): δ 28.6 (NCH_3_), 43.8 (CH_2_), 73.3 (CH), 107.6 (CH), 111.7 (C), 112.0 (CH), 118.7 (C), 126.3 (2 × CH), 131.5 (C), 132.5 (2 × CH), 135.7 (C), 140.9 (C), 149.0 (C), 154.2 (CO). C-NO_2_ was not observed under these experimental conditions. HRMS (EI): calcd for C_17_H_13_N_3_O_5_ (M^+^) 357.1193, found 357.1194.

*5-(2-Hydroxy-2-(2-nitrophenyl)ethyl)-3-methyl-6-nitrobenzo[d]oxazol-2(3H)-one* (**5d**): Brown solid; mp 130 °C; ^1^H-NMR (CDCl_3_): δ 3.36 (dd, *J* = 13.8 Hz, *J* = 8.8 Hz, 1H, CH), 3.41 (s, 3H, NCH_3_), 3.56 (dd, *J* = 13.8 Hz, *J* = 3.2 Hz, 1H, CH), 5.47 (dd, *J* = 8.8 Hz, *J* = 3.2 Hz, 1H, CH), 7.05 (s, 1H, CH), 7.44 (t, *J* = 7.0 Hz, 1H, CH), 7.65 (t, *J* = 7.6 Hz, 1H, CH), 7.73 (s, 1H, CH), 7.80 (d, *J* = 7.0 Hz, 1H, CH), 7.89 (d, *J* = 7.6 Hz, 1H, CH). ^13^C-NMR (CDCl_3_): δ 28.5 (NCH_3_), 40.6 (CH_2_), 70.4 (CH), 107.1 (CH), 111.0 (CH), 124.5 (CH), 128.4 (CH), 128.6 (CH), 131.1 (C), 133.9 (CH), 135.5 (C), 139.1 (C), 140.5 (C), 144.8 (C), 147.4 (C), 154.3 (CO). HRMS (EI): calcd for C_16_H_13_N_3_O_7_ (M^+^) 337.1092, found 337.1092.

*5-(2-(2-Bromophenyl)-2-hydroxyethyl)-3-methyl-6-nitrobenzo[d]oxazol-2(3H)-one* (**5e**): Yellow solid; mp 159 °C; ^1^H-NMR (DMSO-*d*_6_): δ 3.29 (s, 3H, NCH_3_), 3.30–3.33 (m, 2H, CH_2_), 5.51 (bs, 1H, CH), 7.15 (s, 1H, CH), 7.21 (d, *J* = 7.3 Hz, 1H, CH), 7.40 (t, *J* = 7.7 Hz, 1H, CH), 7.51–7.54 (m, 2H, 2 × CH), 7.95 (s, 1H, CH). ^13^C-NMR (DMSO-*d*_6_): δ 28.6 (NCH_3_), 40.5 (CH_2_), 71.35 (CH), 106.6 (CH), 111.9 (CH), 121.4 (C), 128.1 (CH), 128.3 (CH), 129.3 (C), 130.5 (CH), 132.3 (CH), 135.4 (C), 140.1 (C), 143.8 (C), 144.7 (C), 154.3 (CO). HRMS (EI): calcd for C_16_H_13_BrN_2_O_5_ (M^+^) 410.0346, found 410.0347.

*5-(2-(3-Bromophenyl)-2-hydroxyethyl)-3-methyl-6-nitrobenzo[d]oxazol-2(3H)-one* (**5f**): Yellow solid; mp 154 °C; ^1^H-NMR (CDCl_3_): δ 2.15 (d, *J* = 2.9 Hz, 1H, OH), 3.13 (dd, *J* = 13.6 Hz, *J* = 9.0 Hz, 1H, CH), 3.44 (s, 3H, NCH_3_), 3.53 (dd, *J* = 13.6 Hz, *J* = 3.4 Hz, 1H, CH), 5.08 (dd, *J* = 9.0 Hz, *J* = 3.4 Hz, 1H, CH), 6.87 (s, 1H, CH), 7.29 (s, 1H, CH), 7.35–7.48 (m, 2H, 2 × CH), 7.61–7.63 (m, 1H, CH), 7.95 (s,1H, CH). ^13^C-NMR (CDCl_3_): δ 28.5 (NCH_3_), 43.7 (CH_2_), 73.4 (CH), 107.5 (CH), 111.9 (CH), 122.8 (C), 124.3 (CH), 128.7 (CH), 130.2 (CH), 131.0 (CH), 131.7 (C), 135.6 (C), 140.8 (C), 144.0 (C), 146.1 (C), 154.3 (CO). Anal. Calcld for C_16_H_13_BrN_2_O_5_ (393.19) C, 48.88; H, 3.33; N, 7.12. Found: C, 49.11; H, 3.46; N, 7.28.

*Ethyl 2-hydroxy-3-(3-methyl-6-nitro-2-oxo-2,3-dihydrobenzo[d]oxazol-5-yl)propanoate* (**5g**): Yellow solid; mp 136 °C; ^1^H-NMR (CDCl_3_): δ 1.32 (t, *J* = 7.1 Hz, 3H, CH_3_), 2.99 (d, *J* = 5.3 Hz, 1H, OH), 3.17 (dd, *J* = 13.9 Hz, *J* = 8.8 Hz, 1H, CH), 3.46 (s, 3H, NCH_3_), 3.68 (dd, *J* = 13.9 Hz, *J* = 3.7 Hz, 1H, CH), 4.28 (q, *J* = 7.1 Hz 2H, CH_2_), 4.50–4.55 (m, 1H, CH), 7.02 (s, 1H, CH), 7.91 (s, 1H, CH). ^13^C-NMR (CDCl_3_): δ 14.1 (CH_3_), 28.6 (NCH_3_), 37.9 (CH_2_), 62.4 (CH_2_), 70.1 (CH), 107.4 (CH), 111.6 (CH), 130.3 (CH), 135.5 (CH), 140.8 (CH), 144.2 (CH), 154.2 (CO), 173.9 (CO). Anal. Calcld for C_13_H_14_N_2_O_7_ (310.26) C, 50.33; H, 4.55; N, 9.03. Found: C, 50.28; H, 4.54; N, 8.91

*Diethyl 2-hydroxy-2-((3-methyl-6-nitro-2-oxo-2,3-dihydrobenzo[d]oxazol-5-yl)methyl)malonate* (**5h**): Yellow solid; mp 111 °C; ^1^H-NMR (CDCl_3_): δ 1.27 (t, *J* = 7.1 Hz, 6H, 2xCH_3_), 3.43 (s, 3H, NCH_3_), 3.85 (bs, 1H, OH), 3.88 (s, 2H, CH_2_), 4.11–4.34 (m, 4H, CH_2_), 7.14 (s, 1H, CH), 7.75 (s, 1H, CH). ^13^C-NMR (CDCl_3_): δ 13.9 (2 × CH_3_), 28.5 (NCH_3_), 35.5 (2 × CH_2_), 63.1 (CH_2_), 78.4 (C-OH), 107.2 (CH), 111.8 (CH), 127.1 (C), 134.8 (C), 140.8 (C), 145.6 (C), 154.2 (CO), 169.4 (2 × CO). Anal. Calcld for C_16_H_18_N_2_O_9_ (382.32) C, 50.26, H, 4.75, N, 7.33. Found: C, 50.25, H, 4.83, N, 7.18.

*5-((1-Hydroxy-2-oxo-1,2-dihydroacenaphthylen-1-yl)methyl)-3-methyl-6-nitrobenzo[d]oxazol-2(3H)-one* (**5i**): Green solid; mp 204 °C; ^1^H-NMR (CDCl_3_): δ 3.45 (s, 3H, NCH_3_), 3.64 (d, *J* = 14.0 Hz, 1H, CH), 3.84 (d, *J* = 14.0 Hz, 1H, CH), 7.12 (s, 1H, CH), 7.27 (d, *J* = 7.3 Hz, 1H, CH), 7.61 (dd, *J* = 8.0 Hz, *J* = 7.3 Hz,1H, CH), 7.77 (dd, *J* = 7.8 Hz, *J* = 7.3 Hz, 1H, CH), 7.88 (s, 1H, CH), 7.89–7.98 (m, 2H, 2 × CH), 8.15 (d, *J* = 8.0 Hz, 1H, CH). ^13^C-NMR (CDCl_3_): δ 28.6 (NCH_3_), 40.9 (CH_2_), 79.8 (C-OH), 107.5 (CH), 112.4 (CH), 120.3 (CH), 122.7 (CH), 125.9 (CH), 128.6 (CH), 128.7 (CH+C), 130.1 (C), 130.7 (C), 132.4 (CH), 135.3 (C), 138.7 (C), 140.7 (C), 141.0 (C), 144.5 (C), 154.3 (CO); 203.8 (CO). Anal. Calcld for C_21_H_14_N_2_O_6_ (390.35) C, 64.62, H, 3.62, N, 7.18. Found: C, 64.15, H, 3.72, N, 7.05.

*5-((3-Hydroxy-1-methyl-2-oxoindolin-3-yl)methyl)-3-methyl-6-nitrobenzo[d]oxazol-2(3H)-one* (**5j**): Yellow solid; mp 253 °C; ^1^H-NMR (DMSO-*d*_6_): δ 3.03 (s, 3H, NCH_3_), 3.31 (s, 3H, NCH_3_), 3.36 (d, *J* = 13.7 Hz, 1H, CH), 3.66 (d, *J* = 13.7 Hz, 1H, CH), 6.19 (s, 1H, CH), 6.78 (d, *J* = 6.8 Hz, 1H, CH), 6.91–6.95 (m, 2H, 2 × CH), 7.13 (s, 1H, CH), 7.24–7.31 (m, 1H, CH). ^13^C-NMR (DMSO-*d*_6_): δ 26.0 (NCH_3_); 28.5 (NCH_3_), 75.4 (C-OH), 106.7 (CH), 108.6 (CH), 112.9 (CH), 122.3 (CH), 123.9 (CH), 127.6 (C), 129.4 (CH), 130.5 (C), 135.0 (C), 140.3 (C), 142.8 (C), 144.7 (C), 154.3 (CO), 176.8 (CO). C-NO_2_ was not observed under these experimental conditions. Anal. Calcld for C_18_H_15_N_3_O_6_ (369.33) C, 58.54, H, 4.09, N, 11.38. Found: C, 58.26, H, 4.25, N, 11.01.

*1-(3-Methyl-6-nitro-2-oxo-2,3-dihydrobenzo[d]oxazol-5-yl)-2-(4-nitrophenyl)ethyl 4-nitrobenzoate* (**6**): Yellow solid; mp 305 °C; ^1^H-NMR (CDCl_3_): δ 3.35 (s, 3H, NCH_3_), 3.73 (d, *J* = 6.2 Hz, 2H, CH_2_), 6.44 (t, *J* = 6.2 Hz, 1H, CH), 6.84 (s, 1H, CH), 7.68 (d, *J* = 8.5 Hz, 2H, 2 × CH), 7.97 (s, 1H, CH), 8.17 (d, *J* = 8.8 Hz, 2H, 2 × CH), 8.28 (d, *J* = 8.5 Hz, 1H, 2 × CH), 8.32 (d, *J* = 8.5 Hz, 1H, 2 × CH). ^13^C-NMR (CDCl_3_): δ 28.5 (NCH_3_), 40.8 (CH_2_), 76.4 (CH), 108.1 (CH), 110.7 (CH), 114.1 (C), 123.8 (2 × CH), 124.3 (2 × CH), 127.1 (2 × CH), 129.6 (C), 130.7 (2 × CH), 134.4 (C), 135.9 (C), 141.2 (C), 145.9 (C), 148.1 (C), 150.9 (C), 153.8 (CO). HRMS (EI): calcd for C_23_H_16_N_4_O_10_ (M^+^) 526.1205, found 526.1209.

### 3.4. General Procedure for the Reaction of **3** and Aromatic Carbonyl Derivatives **4a**–**f**, α-Carbonyl Ester **4g**, Ketomalonate **4h**, Acenaphtenedione **4i** and Keto-lactam **4j** Using TDAE

A solution of **3** (0.5 g, 1.36 mmol) in anhydrous DMF (10 mL) and the corresponding carbonyl derivative **4a**–**j** (4.098 mmol, 3 equivalents) were placed under nitrogen at −20 °C in a two-necked flask equipped with a silica-gel drying tube and a nitrogen inlet. The solution was stirred and maintained at this temperature for 30 min and then the TDAE (0.48 mL, 2.049 mmol, 1.5 equivalent) was added dropwise via a syringe. A red color immediately developed with the formation of a fine white precipitate. The solution was vigorously stirred at −20 °C for 1 h and then warmed to rt for 2 h. After this time TLC analysis (dichloromethane) clearly showed that **3** was totally consumed. The orange-red turbid solution was filtered (to remove the octamethyloxamidinium dibromide) and hydrolyzed with 80 mL of H_2_O. The aqueous solution was extracted with toluene (3 × 40 mL), the combined organic layers washed with H_2_O (3 × 40 mL) and dried over MgSO_4_. Evaporation of the solvent left an orange viscous liquid as crude product. Purification by silica gel chromatography and recrystallization in ethyl alcohol solvent gave the corresponding oxiranes **7a**–**j**.

*3-Methyl-6-nitro-5-(3-(4-nitrophenyl)oxiran-2-yl)benzo[d]oxazol-2(3H)-one* (**7a**) *trans*-isomer: Yellow solid; mp 224 °C; ^1^H-NMR (CDCl_3_): δ 3.52 (s, 3H, NCH_3_), 3.92 (d, *J* = 1.9 Hz, 1H, CH), 4.54 (d, *J* = 1.9 Hz, 1H, CH), 7.33 (s, H, CH), 7.60 (d, *J* = 8.7 Hz, 2H, 2 × CH), 8.13 (s, 1H, CH), 8.30 (d, *J* = 8.7 Hz, 2H, 2 × CH). ^13^C-NMR (CDCl_3_): δ 28.8 (NCH_3_), 61.0 (CH), 61.1 (CH), 105.7 (CH), 107.4 (CH), 124.0 (2 × CH), 126.6 (2 × CH), 131.8 (C), 137.28 (C), 141.6 (C), 142.1 (C), 143.0 (C), 148.2 (CO). C-NO_2_ was not observed under these experimental conditions. HRMS (EI): calcd for C_16_H_11_N_3_O_7_ (M^+^) 375.0935, found 375.0943.

*5-(3-(4-Bromophenyl)oxiran-2-yl)-3-methyl-6-nitrobenzo[d]oxazol-2(3H)-*one (**7b**) *trans*-isomer: Yellow solid; mp 209 °C; ^1^H-NMR (CDCl_3_): δ 3.51 (s, 3H, NCH_3_), 3.77 (d, *J* = 1.9 Hz, 1H, CH), 3.54 (d, *J* = 1.9 Hz, 1H, CH), 7.28 (d, *J* = 8.4 Hz, 2H, 2 × CH), 7.31 (s, 1H, CH), 7.54 (d, *J* = 8.4 Hz, 2H, 2 × CH), 8.11 (s, 1H, CH). ^13^C-NMR (CDCl_3_): δ 28.7 (NCH_3_), 60.5 (CH), 61.7 (CH), 105.6 (CH), 107.3 (CH), 122.8 (C), 127.5 (2 × CH), 131.9 (2 × CH), 132.5 (C), 134.8 (C), 137.0 (C), 141.3 (C), 154.1 (CO). Anal. Calcld for C_16_H_11_BrN_2_O_5_ (391.17) C, 49.13; H, 2.83; N, 7.16. Found: C, 49.27; H, 2.92; N, 7.85.

*4-(3-(3-Methyl-6-nitro-2-oxo-2,3-dihydrobenzo[d]oxazol-5-yl)oxiran-2-yl)*benzonitrile (**7c**) *trans*-isomer: Yellow solid; mp 213 °C; ^1^H-NMR (CDCl_3_): δ 3.51 (s, 3H, NCH_3_), 3.86 (d, *J* = 1.8 Hz, H, CH), 4.52 (d, *J* = 1.8 Hz, H, CH), 7.32 (s, 1H, CH), 7.53 (d, *J* = 8.3 Hz, 2H, 2 × CH), 7.72 (d, *J* = 8.3 Hz, 2H, 2 × CH), 8.12 (s, 1H, CH). ^13^C-NMR (CDCl_3_): δ 28.7(NCH_3_), 61.0 (CH), 61.2 (CH), 105.7 (CH), 107.3 (CH), 112.6 (C), 118.5 (C), 126.5 (2 × CH), 131.9 (C), 132.5 (2 × CH), 137.1(C), 141.1 (C), 141.5 (C), 142.1(C); 154.1 (CO). HRMS (EI): calcd for C_17_H_11_N_3_O_5_ (M^+^) 355.1037, found 355.1036.

*3-Methyl-6-nitro-5-(3-(2-nitrophenyl)oxiran-2-yl)benzo[d]oxazol-2(3H)-*one (**7d**) *trans*-isomer: yellow solid; mp 215 °C; ^1^H-NMR (CDCl_3_): δ 3.52 (s, 3H, NCH_3_), 4.54 (d, *J* = 2.0 Hz, 1H, CH), 4.60 (d, *J* = 2.0 Hz, 1H, CH), 7.36 (s, 1H, CH), 7.52–7.61 (m, 1H, CH), 7.75–7.77 (m, 2H, 2 × CH), 8.14 (s, 1H, CH), 8.23 (d, *J* = 8.0 Hz, 1H, CH). ^13^C-NMR (CDCl_3_): δ 28.7 (NCH_3_), 59.9 (CH), 60.0 (CH), 105.5 (CH), 107.6 (CH), 125.2 (CH), 126.9 (CH), 129.2 (C), 131.8 (CH), 132.5 (C), 134.4 (CH), 137.0 (C), 141.5 (C), 142.6 (C), 147.9 (C), 154.2 (CO). HRMS (EI): calcd for C_16_H_11_N_3_O_7_ (M^+^) 375.0935, found 375.0940.

*3-Methyl-6-nitro-5-(3-(2-nitrophenyl)oxiran-2-yl)benzo[d]oxazol-2(3H)-one* (**7d**) *cis*-isomer: Beige solid; mp 166 °C; ^1^H-NMR (CDCl_3_): δ 3.38 (s, 3H, NCH_3_), 5.14 (d, *J* = 4.9 Hz, 1H, CH), 5.17 (d, *J* = 4.9 Hz, 1H, CH), 7.03 (s, 1H, CH), 7.30–7.40 (m, 1H, CH), 7.44–7.46 (m, 2H, 2 × CH), 7.86–7.90 (m, 2H, 2 × CH). ^13^C-NMR (CDCl_3_): δ 28.6 (NCH_3_), 59.0 (2 × CH), 107.3 (CH), 107.4 (CH), 124.7 (CH), 128.8 (CH), 128.9 (C), 129.2 (CH), 129.4 (C), 132.7 (CH), 135.9 (C), 141.2 (C), 148.5 (C), 153.9 (CO). Anal. Calcld for C_16_H_11_N_3_O_7_ (357.27) C, 53.79; H, 3.10; N, 11.76. Found: C, 53.48; H, 3.30; N, 11.44.

*5-(3-(2-Bromophenyl)oxiran-2-yl)-3-methyl-6-nitrobenzo[d]oxazol-2(3H)-one* (**7e**) *trans*-isomer: Green solid; mp 203 °C; ^1^H-NMR (CDCl_3_): δ 3.52 (s, 3H, NCH_3_), 4.09 (d, *J* = 1.9 Hz, 1H, CH), 4.57 (d, *J* = 1.9 Hz, 1H, CH), 7.20–7.29 (m, 1H, CH), 7.36 (s, 1H, CH), 7.38–7.47 (m, 2H, 2 × CH), 7.60 (d, *J* = 7.5 Hz, 1H, CH), 8.12 (s, 1H, CH). ^13^C-NMR (CDCl_3_): δ 28.7 (NCH_3_), 60.1 (CH), 62.0 (CH), 105.6 (CH), 107.4 (CH), 123.1 (C), 126.1 (CH), 127.8 (CH), 130.0 (CH), 132.2 (C), 132.7 (CH), 135.2 (C), 137.0 (C), 141.4 (C), 142.4 (C), 154.2 (CO). Anal. Calcld for C_16_H_11_BrN_2_O_5_ (391.17) C, 49.13; H, 2.83; N, 7.16. Found: C, 49.27; H, 2.93; N, 7.17.

*5-(3-(2-Bromophenyl)oxiran-2-yl)-3-methyl-6-nitrobenzo[d]oxazol-2(3H)-one* (**7e**) *cis*-isomer: Green solid; mp 151 °C; ^1^H-NMR (CDCl_3_): δ 3.42 (s, 3H, NCH_3_), 4.71 (d, *J* = 4.4 Hz, 1H, CH), 5.17 (d, *J* = 4.4 Hz, 1H, CH), 6.98–7.14 (m, 3H, 3 × CH), 7.16 (s, 1H, CH), 7.36–7.42 (m, 1H, CH), 7.93 (s, 1H, CH). ^13^C-NMR (CDCl_3_): δ 28.6 (NCH_3_), 59.3 (CH), 61.0 (CH), 107.3 (CH), 107.9 (CH), 122.5 (C), 126.4 (CH), 128.0 (CH), 129.4 (C), 129.6 (CH), 132.7 (CH), 132.9 (C), 135.8 (C), 141.1 (C), 142.5 (C), 154.0 (CO). Anal. Calcld for C_16_H_11_BrN_2_O_5_ (391.17) C, 49.13; H, 2.83; N, 7.16. Found: C, 49.42; H, 3.02; N, 7.28.

*5-(3-(3-Bromophenyl)oxiran-2-yl)-3-methyl-6-nitrobenzo[d]oxazol-2(3H)-one* (**7f**) *trans*-isomer: Beige solid; mp 165 °C; ^1^H-NMR (CDCl_3_): δ 3.51 (s, 3H, 3H, NCH_3_), 3.77 (d, *J* = 1.9 Hz, 1H, CH), 4.55 (d, *J* = 1.9 Hz, 1H, CH), 7.24–7.28 (m, 1H, CH), 7.32 (s, 1H, CH), 7.33–7.38 (m, 1H, CH), 7.49–7.54 (m, 2H, 2 × CH), 8.11 (s, 1H, CH). ^13^C-NMR (CDCl3): δ 28.7 (NCH_3_), 60.6 (CH), 61.4 (CH), 105.7 (CH), 107.3 (CH), 122.8 (C), 124.6 (CH), 128.7 (CH), 130.2 (CH), 131.9 (CH), 132.3 (C), 137.0 (C), 138.1 (C), 141.4 (C), 142.1 (C), 154.1 (CO). Anal. Calcld for C_16_H_11_BrN_2_O_5_ (391.17) C, 49.13; H, 2.83; N, 7.16. Found: C, 49.30; H, 2.97; N, 7.10.

*Ethyl 3-(3-methyl-6-nitro-2-oxo-2,3-dihydrobenzo[d]oxazol-5-yl)oxirane-2-carboxylate* (**7g**) *trans*-isomer: Light yellow needles; mp 199 °C; ^1^H-NMR (CDCl_3_): δ 1.36 (t, *J* = 7.2 Hz, H, CH); 3.38 (d, *J* = 1.9 Hz, 3H, CH); 3.48 (s, 3H, NCH_3_); 4,35 (q, *J* = 7.2 Hz, 2H, CH_2_); 4,75 (d, *J* = 1.9 Hz, 1H, CH); 7.19 (s, 1H, CH); 8.12 (s, 1H, CH). ^13^C-NMR (CDCl_3_): δ 14.1 (CH_3_), 28.8 (NCH_3_), 56.0 (CH), 56.6 (CH), 62.2 (CH_2_), 105.8 (CH), 107.4 (CH), 130.9 (C), 137.0 (C), 141.6 (C), 142.2 (C), 154.0 (CO), 167.2 (CO). HRMS (EI): calcd for C_13_H_12_N_2_O_7_ (M^+^) 309.0717, found 309.0713.

*Diethyl 3-(3-methyl-6-nitro-2-oxo-2,3-dihydrobenzo[d]oxazol-5-yl)oxirane-2,2-dicarboxylate* (**7h**) *trans*-isomer: Dark brown; mp 118 °C; ^1^H-NMR (CDCl_3_): δ 0.98 (t, *J* = 7.2 Hz, 3H, CH_3_), 1.37 (t, *J* = 7.2 Hz, 3H, CH_3_), 3.48 (s, 3H, NCH_3_), 3.98 (q, *J* = 7.2 Hz, 2H, CH_2_), 4.39 (q, *J* = 7.2 Hz, 2H, CH_2_), 5.14 (s, 1H, CH), 7.24 (s, 1H, CH), 8.12 (s, 1H, CH). ^13^C-NMR (CDCl_3_): δ 13.8 (CH_3_), 14.0 (CH_3_), 28.9 (NCH_3_), 61.2 (CH), 62.2 (CH_2_), 63.3 (CH_2_), 107.2 (CH), 107.4 (CH), 127.9 (C), 136.7 (C), 141.9 (C), 142.3 (C), 153.9 (C), 163.3 (CO), 164.6 (CO). Anal. Calcld for C_16_H_16_N_2_O_9_ (380.31) C, 50.53; H, 4.24; N, 7.37. Found: C, 50.96; H, 4.54; N, 7.25.

*3-Methyl-6-nitro-5-(2-oxo-2H-spiro[acenaphthylene-1,2'-oxiran]-3'-yl)benzo[d]oxazol-2(3H)-one* (**7i**) *like*-isomer: Yellow solid; mp 235 °C; ^1^H-NMR (CDCl_3_): δ 3.59 (s, 3H, NCH_3_), 5.29 (s, 1H, CH), 7.62 (d, *J* = 6.8 Hz, 1H, CH_2_), 7.74 (s, 2H, 2 × CH); 7.77–7.80 (m, 1H, CH), 7.85 (d, *J* = 6.7 Hz, 1H, CH_2_), 8.01 (d, *J* = 8.4 Hz, 1H, CH_2_), 8.06 (s, 1H, CH), 8.19 (d, *J* = 8.1 Hz, 1H, CH). ^13^C-NMR (CDCl_3_): δ 28.9 (NCH_3_), 65.8 (CH), 67.1 (C), 106.8 (CH), 108.9 (CH), 118.9 (CH), 122.1 (CH), 126.5 (CH), 128.3 (CH), 128.7 (CH), 129.2 (C), 130.4 (C), 131.2 (C), 132.1 (C), 132.3 (CH), 136.5 (C), 141.4 (C), 141.7 (C), 142.5 (C), 154.2 (CO), 196.0 (CO). Anal. Calcld for C_21_H_12_N_2_O_6_ (388.33) C, 64.95; H, 3.11; N, 7.21. Found: C, 64.08; H, 3.26; N, 6.85.

*3-Methyl-6-nitro-5-(2-oxo-2H-spiro[acenaphthylene-1,2'-oxiran]-3'-yl)benzo[d]oxazol-2(3H)-one* (**7i**) *unlike*-isomer: Beige solid; mp 201 °C; ^1^H-NMR (CDCl_3_): δ 3.63 (s, 3H, NCH_3_), 5.30 (s, 1H, CH), 6.34 (d, *J* = 6.8 Hz, 1H, CH_2_), 7.32 (d, *J* = 6,7 Hz, 1H, CH), 7.67 (s, 1H, CH), 7.77–7.88 (m, 2H, 2 × CH), 8.07 (s, 1H, CH), 8.12 (d, *J* = 1.7 Hz,1H, CH), CH), 8.16 (d, *J* = 3.2 Hz,1H, CH). ^13^C-NMR (CDCl_3_): δ 29.0 (NCH_3_), 64.7 (CH), 66.5 (C); 107.3 (CH), 107.7 (CH), 119.1 (CH), 122.7 (CH), 126.8 (CH), 127.8 (CH), 128.5 (CH), 129.7 (C), 130.2 (C), 130.5 (C), 130.6 (C), 132.3 (CH), 136.9 (C), 141.9 (C), 143.2 (C), 154.0 (C), 196.3 (CO). C-NO_2_ was not observed under these experimental conditions. HRMS (EI): calcd for C_21_H_12_N_2_O_6_ (M^+^) 389.0768, found 389.0768.

*1-Methyl-3'-(3-methyl-6-nitro-2-oxo-2,3-dihydrobenzo[d]oxazol-5-yl)spiro[indoline-3,2'-oxiran]-2-one* (**7j**) *like*-isomer: Beige solid; mp 190 °C; ^1^H-NMR (CDCl_3_): δ 3.13 (s, 3H, NCH_3_), 3.54 (s, 3H, NCH_3_), 5.15 (s, 1H, CH), 6.93 (dd, *J* = 7.8 Hz, *J* = 0.7 Hz,1H, CH), 7.17 (td, *J* = 7.5 Hz, *J* = 0.7 Hz,1H, CH), 7.32 (dd, *J* = 7.3 Hz, *J* = 0.7 Hz, 1H, CH), 7.44 (td, *J* = 7.3 Hz, *J* = 1.4 Hz, 1H, CH), 7.62 (s, 1H, CH), 8.08 (s, 1H, CH). ^13^C-NMR (CDCl_3_): δ 26.5 (NCH_3_), 28.8(NCH_3_), 62.7 (CH), 65.0 (C), 106.8 (CH), 108.9 (CH), 109.0 (CH), 122.2 (CH), 122.3 (C), 123.2 (CH), 128.8 (C), 130.8 (CH), 136.4 (C), 141.5 (C), 141.7 (C), 144.8 (CH), 154.2 (CO), 169.6 (CO). Anal. Calcld for C_18_H_13_N_3_O_6_ (367.31) C, 58.86; H, 3.57; N, 11.44. Found: C, 58.85; H, 3.71; N, 11.31.

*1-Methyl-3'-(3-methyl-6-nitro-2-oxo-2,3-dihydrobenzo[d]oxazol-5-yl)spiro[indoline-3,2'-oxiran]-2-one* (**7j**) *unlike*-isomer: Beige solid; mp 211 °C; ^1^H-NMR (CDCl_3_): δ 3.33 (s, 3H, NCH_3_), 3.59 (s, 3H, NCH_3_), 5.18 (s, 1H, CH), 6.01 (d, *J* = 7.5 Hz, 1H, CH), 6.71 (t, *J* = 7.5 Hz, 1H, CH), 6.88 (d, *J* = 7.7 Hz, 1H, CH), 7.32 (d, *J* = 7.7 Hz, 1H, CH), 7.57 (s, 1H, CH), 8.08 (s, 1H, CH). ^13^C-NMR (CDCl_3_): δ 26.8 (NCH_3_), 29.0 (NCH_3_), 62.2 (CH), 64.4 (C), 107.3 (CH), 107.8 (CH), 109.2 (CH), 119.8 (C), 122.0 (CH), 122.4 (CH), 129.4 (C), 130.9 (CH), 137.0 (C), 141.8 (C), 141.9 (C), 145.6 (C), 154.0 (CO), 170.6 (CO). HRMS (EI): calcd for C_18_H_13_N_3_O_6_ (M^+^) 368.0877, found 368.0876.

## 4. Conclusions

In conclusion, we have investigated the reactivity of some new benzoxazolone derivatives formed via the TDAE strategy. This is the first example of the use of the TDAE strategy to generate a benzoxazolinonic anion, which cannot be formed via the standard organometallic strategy. This study brought to light a new and original reactivity and we have defined some limitations of the TDAE strategy. We show that 5-(bromomethyl)-3-methyl-6-nitrobenzo[*d*]oxazol-2(3*H*)-one (**2**), in addition to providing the expected alcohols **5a**–**i** in moderate to good yields, furnished an unexpected ester **6** formed in 23% yield, particularly with the *p*-nitrobenzaldehyde. The reactions of 5-(dibromomethyl)-3-methyl-6-nitro-benzo[*d*]oxazol-2(3*H*)-one (**3**) led to the expected oxiranes **7a**–**j** and mixtures of original stereoisomers **7i**–**j** in good yields. All these synthesized products are currently undergoing pharmacological evaluation.
